# Prevalence of human immunodeficiency virus, syphilis, hepatitis B and C in blood donations in Namibia

**DOI:** 10.1186/1471-2458-14-424

**Published:** 2014-05-05

**Authors:** Rooyen T Mavenyengwa, Munyaradzi Mukesi, Israel Chipare, Esra Shoombe

**Affiliations:** 1 Department of Health Sciences, School of Health and Applied Sciences, Polytechnic of Namibia, Private Bag 13388, Windhoek, Namibia; 2 Namibia Blood Transfusion Services, P.O. Box 3434, Windhoek, Namibia

## Abstract

**Background:**

Transfusion Transmissible Infections (TTIs) such as Human Immunodeficiency Virus (HIV), syphilis, hepatitis B virus (HBV) and hepatitis C virus (HCV) are infections which are common in some communities in Southern Africa. It is important to screen blood donations for these infections.

**Methods:**

This is a retrospective study which involved reviewing of previous blood donation records for the year 2012 in Namibia. The records were analyzed to determine the prevalence of HIV, syphilis, Hepatitis B and C among blood donations with regard to gender, age and geographical region of the donors.

**Results:**

The findings indicated a significantly low prevalence of HIV, syphilis, HBsAg and anti-Hepatitis C among the blood donations. A low infection rate of 1.3% by any of the four tested TTIs was found among the blood donations given by the donor population in Namibia in 2012.

**Conclusion:**

The blood donations given by the donor population in Namibia has a low infection rate with the HIV, syphilis, HBsAg and anti-HCV. A strict screening regime must continue to be used as the infections are still present albeit in small numbers.

## Background

Blood transfusion is a source of transfusion transmissible infections (TTIs) especially caused by bacteria or viruses. These are transmitted through transfusion of different blood products from blood donors to recipients. Transfusion-associated infections, mainly hepatitis B virus (HBV), hepatitis C virus (HCV), human immunodeficiency virus (HIV) and syphilis among blood donors are of public health concern. Blood transfusion practices worldwide emphasize on safety and protection of human life. Infections such as HIV, HBV, HCV are of great concern because of their prolonged viraemia and carrier or latent state [1]. These are routinely tested on all donated blood in many parts of the Africa where each unit of blood collected undergoes strict screening tests to ensure safety to recipients [2].

Blood transfusion services offered by the Namibia Transfusion Services (NAMBTS) remain a vital and necessary part of the Namibia health care sector. It is an integral part of patient treatment through administration of different blood products to those in need. Proper testing and screening of these TTIs among blood and platelet donors permits an assessment of the blood donor population to ensure safety of the collected donations. It is necessary to estimate the prevalence of these infections in the Namibian donor community using more sensitive and specific tests than the early tests used [3-5].

The battery of screening tests conducted on donor blood has substantially reduced the risk of TTIs, although it has increased the cost of providing safe blood [6]. Screening of potential blood donors has historically relied on the use of immunoassays to detect viral antibodies or antigens. Currently, besides serological tests, prevention of transfusion associated infection depends on use of molecular tests [7,8]. Proper pre-donation selection of donors, followed by serologic testing for infectious pathogens, including HBV, HCV and HIV is also necessary [6]. Since 1999, new screening methods involving nucleic acid amplification testing (NAT) to detect HIV type 1 and HCV Ribonucleic acid (RNA) were approved by the Food and Drug Administration [9].

Studies have been done in Namibia to determine prevalence of TTIs such as hepatitis B and hepatitis C viruses among blood donors [3-5]. Insensitive countercurrent electrophoresis, was used among members of the Kwangali tribe in the west [3]. In another early study the HBsAg carrier rate was found to be slightly higher but less sensitive methods were used [10]. The varying frequency of the HBsAg prevalence can be attributed to many factors, including the socio-economic conditions in which the people live, the age, sex distribution, genetic make-up of the population and the risk of exposure to blood-sucking vectors [4]. There is need to investigate socio-demographic characteristics that are associated with TTIs. This should include categorization of donors into regular, first time and occasional donors. Hepatitis C virus is an important TTI whose infection leads to long term sequelae including cirrhosis, liver failure and hepatocellular carcinoma [5]. Hepatitis C virus data from southern Africa are incomplete since no survey has been published for countries like Angola and Namibia. Countries in central and southern Africa show a range of HCV prevalence from <1% to >10% [5]. Recent review of prevalence of HCV globally based on the number of people with anti-HCV, has shown that these increased from 2.3% to 2.8% and from more than 122 million to more than 185 million between 1990 and 2005 and are therefore at risk of developing liver cirrhosis and liver cancer [11].

The prevalence of HIV infection in Africa varies from one region to another. In South Africa and Central African Republic it was respectively 0.10 to 15% in new donors in 2004 [12]. The prevalence rates of HIV-1 decreased after implementation of safety policies to screen HIV from donated blood [13]. Despite the screening of blood products, blood transfusion still accounts for 5 to 10% of HIV infection in Sub-Saharan Africa, similarly 5% to 12% of patients who received blood transfusion developed hepatitis [1]. Like HIV, syphilis is still one of the infections transmitted through blood transfusion as shown by a high antibody detection in healthy African blood [14]. In sub-Saharan Africa, syphilis remains a serious public health problem. Prevalence of active syphilis infection among African countries showed 12.8% in Tanzania, and 3.8% in Kenya [15,16]. The high prevalence of HIV, HBV, HCV and syphilis has heightened the problems of blood safety in some parts of Africa [1]. Screening of blood products has led to a decline in the incidence of acute hepatitis C since the late 1980s, although rates have reached a plateau in recent years [17].

Screening of blood bank donors for HBsAg does not totally eliminate the risk of HBV infection since the absence of this marker in the serum does not exclude the presence of HBV DNA. Therefore, Nucleic Acid Testing (NAT) blood screening is the key that is originally intended to complement serological screening for detection of viruses. The main advantage of NAT screening is detection cases during the window period [18]. More sensitive tests for viral antibodies, antigens and nucleic acids have led to dramatic TTIs and should be used [7].

The study focused on determining the prevalence of TTIs namely hepatitis B and C, HIV and syphilis among blood donations in Namibia from 2 January to 31 December for the year 2012 using a combination of traditional serological techniques and newer molecular methods. It also investigated the existence of any association between the TTIs and socio-demographic characteristics of the donors who contributed the donations.

## Methods

### Study design

This study is a retrospective study, analyzing blood donations from donor records for the year 2012. From the pool of blood donations received by Namibia Blood Transfusion Services (NAMBTS), these came from new, lapsed and regular donors. Lapsed donors were those who had donated previously but had gone for 6 months without any donation. The study was conducted at the NAMBTS headquarters in Windhoek, which is the main provider of blood transfusion services in Namibia and included some repeat donations.

### Study population

The study included 24 761 blood donations from nine regions of Namibia. These blood donations were all from voluntary donors mainly selected based on a set criterion of their age and weight. The individuals were those who weighed not less than 50 kg and were within the age of 16 to 65 years. A blood donor medical history form was filled in by the donor before donating blood. It was used for further donor selection according to the NAMBTS protocol for selecting blood donors.

### Blood collection

Venous blood was collected from the blood donor in blood collection tubes upon donating blood. The blood was used to screen for HIV, syphilis, HBsAg and anti-HCV.

### Serology

The blood was screened on the Abbott Prism for Anti-HIV 1/2, HBsAg and anti-HCV and TPHA for syphilis on the Olympus PK2700. Every reactive result was tested twice and if both replicates are nonreactive, then the initial reactive was interpreted as false positive. Syphilis was tested using automated Treponema pallidum Haemaglutination Assay (TPHA) which when reactive twice was then repeated using the manual TPHA and VDRL test procedure as a confirmation as the latter is regarded as more specific and gave the final screen outcome.

### Nucleic acid testing

The three TTIs HBV, HCV and HIV were tested by means of Nucleic Acid Testing (NAT) using the Chiron Procleix Ultrio Plus Assay, Norvatis, USA. This is a molecular discriminatory assay for HBV, HCV and HIV. All NAT reactive results were replicated and then discriminated to identify the virus. If an initial reactive was not repeat reactive by one or both replicates, this was considered a false positive result and any repeat reactive NAT that could not be discriminated was considered to be discordant and repeat samples collected from the donor after 56 days for retesting.

The algorithm used was such that negative results from serology or NAT were interpreted concurrently and both had to be negative to consider the blood donation as negative for the tested infectious agents and similarly if both were positive the final result was considered positive.

Where there was discordant virology with serology being reactive, the following confirmatory tests were performed; Immunocomb for anti-HIV only if the NAT was negative; ElecsysHBsAg neutralization only if the NAT was negative; and Murex anti-HCV only if the NAT was negative. A NAT repeat reactive with a nonreactive serology was considered a window period case, however no further testing was done to identify occult HBV (OBI).

### Statistical analysis

All data were entered into Microsoft Excel spreadsheets and analyzed using SPSS (version 21, IBM Corp., Armonk, NY, USA). The results of each test were retrieved and used to determine the prevalence of each of the four TTIs included in this study. It was then analyzed based on gender, age and geographical regions from where blood donors from which the donations came from. Pearson’s χ2 tests or Fisher’s exact tests (when values were lower than 5) were used for categorical data. Bivariate analyses assessed associations between population characteristics and TTIs in the data. Odds ratios (OR) and 95% confidence intervals (CI) were calculated for all associations. P values less than 0.05 were considered statistically significant.

### Ethical considerations

The permission to conduct this study was granted by the Director of NAMBTS. In addition the ethics committee within NAMBTS gave ethical approval for the study to be conducted. With regard to confidentiality no names were involved in the data analysis process as only codes were used to identify donors. There was no need for blood donor consent for this study since the study did not directly involve donors but rather the donation units which were not linked to the donor identity.

## Results

A total of 24761 blood donations were retrieved and analyzed into different categories. The donations were tested for four different TTIs.

Table 1 shows the prevalence of the four tested TTIs based on a positive or negative outcome among the donor population considered in the study. The table shows the results of the 24761 blood donations, 316 (1.3%) donations tested positive for HIV, syphilis, HBsAg and anti-HCV. The remainder (24445 donations) tested negative for any of the four tested TTIs. It also showed that HBsAg positive cases were the most common TTIs among the positive donations and HCV was the least positive among these donations. There were 3 (1.8%) cases of HBV and HCV co-infection and 2 (0.9%) cases of HBV and HIV co-infection and only 1 (1.0%) case of HIV and HCV co-infection. These co-infections were overally 0.024% of all the donations.

**Table 1:** Prevalence of TTIs among the general blood donor population of Namibia

	**Number of donations and percentage**				
**Parameter**	**Total sample**	**HIV**	**Syphilis**	**HBsAg**	**HCV**
		**No. (%)**	**No. (%)**	**No. (%)**	**No. (%)**
Positive	316 (1.3)	75 (0.3)	75 (0.3)	140 (0.6)	26 (0.1)
Negative	24445 (98.7)	24686 (99.7)	24686 (99.7)	24621 (99.4)	24735 (99.9)
Total	24761	24761	24761	24761	24761

The gender distribution of donors who contributed the donations is summarized in Table 2. Donations with a positive result for the tested TTIs are categorized based on the gender of the donors to show the prevalence of the TTIs among males and females.

**Table 2:** Prevalence of TTIs among blood donors according to gender

	**Number of donations and percentage**				
**Parameter**	**Total sample**	**HIV**	**Syphilis**	**HBsAg**	**HCV**
		**No. (%)**	**No. (%)**	**No. (%)**	**No. (%)**
Females	11707	37 (0.3)	49 (0.4)	51 (0.4)	11 (0.1)
Males	13054	38 (0.3)	26 (0.2)	89 (0.7)	15 (0.1)
Total	24761	75 (0.3)	75 (0.3)	140 (0.6)	26 (0.1)

A slightly higher number of the donations were from males than from females. There was no significant difference between donations from males and females positive for HIV. Both genders also had a similar HCV positivity. However HBsAg positivity was slightly higher in males than in females by 0.3%. There was a significant difference in infection with syphilis with 0.4% and 0.2% between females and males infected respectively (OR, 2.11; 95% CI, 1.31-93.39; p = 0.002).

Table 3 shows prevalence of TTIs among blood donations according to age groups. Age groups of donors were categorized into age intervals of ten years ranging from donors ≤20 years of age to donors ≥51 years of age.

**Table 3:** Prevalence of TTIs among blood donors according age groups

	**Number of donations positive and percentage**				
**Age group**	**Total sample**	**HIV**	**Syphilis**	**HBsAg**	**HCV**
		**No. (%)**	**No. (%)**	**No. (%)**	**No. (%)**
**≤20**	4501	18 (0.4)	6 (0.1)	38 (0.8)	9 (0.2)
**21-30**	7217	22 (0.3)	9 (0.1)	55 (0.8)	6 (0.1)
**31-40**	5958	17 (0.3)	26 (0.4)	22 (0.4)	4 (0.1)
**41-50**	4171	12 (0.3)	26 (0.6)	19 (0.5)	3 (0.1)
**≤51**		6 (0.2)	8 (0.3)	6 (0.2)	4 (0.1)
**Total**	24761	75 (0.3)	75 (0.3)	140 (0.6)	26 (0.1)

Most donations came from the 21-30 years age group. Age groups of 21-30, 31-40 and 41-50 all had the same percentage of HIV positive donors. Donations from those aged more than 51 years old were the least positive for HIV. For syphilis both age groups of ≤20 and 21-30 years had the least proportion of those testing positive. An analysis of donations from those aged between the age of 46-55 showed that these were significantly infected with syphilis compared to all other age groups (OR, 5.70; 95% CI, 1.34-24.15; p = 0.018). Both age groups of donors who were ≤20 and 21-30 years, had the same percentage of 0.8% positive for HBsAg, the highest among all the four TTIs. Donations from those above 51 years were the least positive for HBsAg. The percentage of those who tested positive for anti-HCV was the same for all age groups except those less than 21 years which was twice as much as the other age groups.

Blood donations from different regions of Namibia are presented in Table 4 to show the prevalence of TTIs among blood donations in those geographical regions.

**Table 4:** Prevalence of TTIs among blood donors according regional location

		**Number of donations positive and percentage**			
**Region**	**Total sample**	**HIV**	**Syphilis**	**HBsAg**	**HCV**
		**No. (%)**	**No. (%)**	**No. (%)**	**No. (%)**
Erongo	4454	17 (0.4)	9 (0.2)	25 (0.6)	2 (0.04)
Hardap	789	0 (0.0)	1 (0.1)	3 (0.4)	1 (0.13)
Karas	668	1 (0.1)	2 (0.3)	10 (1.5)	1 (0.15)
Khomas	15276	45 (0.3)	45 (0.3)	66 (0.4)	19 (0.12)
Kunene	202	0 (0.0)	1 (0.5)	2 (1.0)	0 (0.00)
Omaheke	447	0 (0.0)	3 (0.7)	7 (1.6)	0 (0.00)
Oshana	250	1 (0.1)	5 (2.0)	6 (2.4)	0 (0.00)
Oshikoto	746	6 (0.8)	0 (0.0)	10 (1.3)	1 (0.13)
Otjozondjupa	1929	5 (0.3)	9 (0.5)	11 (0.6)	2 (0.10)
Total	24761	75 (0.3)	75 (0.3)	140 (0.6)	26 (0.1)

The findings indicated that Oshikoto region had the highest percentage of positive donations for HIV while Hardap, Kunene and Omaheke regions showed no positive HIV donations. Oshana region had the highest donations testing positive for both syphilis and HBsAg. Donations from this region were significantly positive for syphilis than any other region (OR, 4.37; 95% CI, 1.45-13.13; p = 0.009). Infection with HCV was the least positive among blood donations in all regions as it was below 0.2%.

Table 5 summarizes the different types of donors who contributed to the donations whilst Table 6 shows the donations that were positive for different TTIs based on donor types. It is apparent that most positive donations were coming from new donors irrespective of the type of TTI.

**Table 5:** Summary of donations based on donor type

**Donor category**	**Number of donors**	**Donations made**	**Donation frequency/yr**
New	3928	3928	1
Lapsed	2840	2840	1
Repeat	5988	17993	3
Total	12756	24761	1.9

**Table 6:** TTIs reactive donations by donor types

**Donor category**	**Anti-HCV reactive**	**Anti-HIV reactive**	**HBsAg reactive**	**TPHA reactive**	**Total**
	**No. (%)**	**No. (%)**	**No. (%)**	**No. (%)**	**No. (%)**
New	13 (48.1)	35 (46.7)	117 (84.2)	39 (52.0)	204 (64.6)
Lapsed	5 (18.5)	16 (21.3)	8 (5.8)	16 (21.3)	45 (14.2)
Repeat	9 (33.3)	24 (32.0)	14 (10.1)	20 (26.7)	67 (21.2)
**Total**	**27**	**75**	**139**	**75**	**316**

## Discussion

This study showed that 1.3% of the donations included in the study were for at least one of the four TTIs. This was relatively a low prevalence when compared to other studies conducted in other African countries such as Tanzania with 15.9% [19] and Ethiopia with 9.5% [3] of blood donors positive for at least one of the mentioned TTIs. According to a report by the Ministry of Health and Social Services (MoHSS) in 2005, HIV and Hepatitis B infection caused blood to be discarded in 7% of blood donations. The prevalence of other screened infections by syphilis and Hepatitis C were 2% [20] indicating that HIV and Hepatitis B had been the most prevalent TTIs among blood donors.

In comparison to the general prevalence of TTIs among donor populations of other African countries, it can be clearly stated that blood donors in Namibia are relatively healthier than other African blood donor populations with regard to the TTIs mentioned. The prevalence found in the donor population may not be the most appropriately suitable for comparison with the general population due to blood donor selection procedures involved in selecting donors. Blood donors undergo pre-donation screening for risk factors, which is aimed to exclude donors potentially infected or those at risk of being infected with TTIs. This may be the reason for the lower prevalence of all four TTIs analyzed in this study.

Prevalence of HIV and syphilis among the donor population was 0.3% for both TTIs. These findings are similar with those presented by the Centre for Disease Control and Prevention (CDC) weekly reported dated 25 November 2011. The reported indicated that the HIV prevalence among Namibian blood donors between the year 2003 to 2008 ranged from 0.3% to 0.7% [21]. This means there has been little variation of HIV prevalence among blood donors during the period of years indicated.

The 0.3% of syphilis positive donations among Namibian donors was very low compared to other findings of studies done in other African countries. In Ghana, 7.5% of blood donors were positive for syphilis in year 2003 [14]. In Ethiopia, 1.8% of blood donors was found to be positive for syphilis [1], and another study conducted in Tanzania found 4.7% syphilis positive donors [19]. The lower prevalence of syphilis in Namibia in comparison to other countries may be caused by factors related to different blood donor sampling procedures used in those studies.

Hepatitis B was the most prevalent TTI with 0.6% of the blood donations positive for HBsAg. This may be significantly low among the donor population but it may also indicate significance among the general population. According to World Health Organization, in year 2006 about 243700 Namibians were found positive with Hepatitis B infections which accounted for 10% of the entire population of Namibia [22]. Infection with HCV was found to be the least with 0.1% donors positive and this was different from the infections rates found in 1999 which indicated 0.9% HCV positive blood donors [5]. This implies that there have been decreases in HCV infections among blood donors which may be possibly caused improved donor selection protocols or alternatively a decrease of HCV among the general population.

The similarity of HIV prevalence among male and female (both 0.3%) blood donations may be due to the reason that both genders are at risk of this infection in a similar manner which mainly unsafe sexual activities. This similarity can also be reflected in other studies from other region which also showed no significant difference of HIV prevalence among male and females. In Tanzania, male blood donors were accounted for 3.8% while female blood donors accounted for 4.0% [19]. The prevalence of syphilis among male and female donors does however show a difference in infection rate. Females were found with higher positive percentage compared to males. Syphilis is generally known to be more prevalent in females rather than in males in Namibia although there are no studies support and explain this observation. In other studies, the ratio of male to female positive for syphilis was reported as 1.4%:0.9% in Ethiopia and 4.8%:4.0% in Tanzania [1,19].

Prevalence of hepatitis B showed a significant difference among male and females with 0.7% males and 0.4% females positive. However HCV infection rate was not different between males and females. There may be no apparent specific factors attributed to these findings but other studies also reported the similar prevalence differences among genders. In Tanzania males were more positive for Hepatitis B than female with a ratio of males to females as 9.1%:6.3%, HCV was reported with a ratio of 1.5%:1.1% [19].

The prevalence of HIV in donations in those aged in the range ≤20 years to donors of 50 years old was between 0.3 to 0.4 which was higher compared to donors above the age of 51 years. The age group with a higher HIV prevalence is consistent with people who are more sexually active than other age groups thus increasing their risk of contracting HIV through unsafe sexual practices. According to the national data provided by the MoHSS, HIV prevalence is increased among people between the ages of 20 to 40 years and decreased among people above the age of 50 years similar to the findings presented in this study. Similar findings were also reported in Tanzania where higher prevalence of HIV was recorded among blood donor of the same ages indicated [19].

The prevalence of TTIs among different regions in Namibia varies from among different regions although the prevalence was low across different regions. Oshikoto region was found with the highest prevalence for HIV positive blood donations. According to the National data of pregnant women in Namibia provided by the MoHSS in 2005 and 2006, Oshana region had the highest prevalence (21%), Oshikoto region (19%) followed by both Omusati and Khomas (16%) regions and the lowest being Kunene region standing at 6% [20]. These findings may however not precisely reflect the prevalence of the blood donor population due donor selection processes involved.

Oshana region had the highest prevalence of syphilis and hepatitis B (2.0% and 2.4% respectively) among all other regions. There may be no other studies conducted to support these findings of increased prevalence of syphilis in Oshana region. With regard to increased prevalence of hepatitis B, this infection is easily transmitted in common activities especially in poor sanitation areas such as those found in Oshana region and other regions in northern Namibia. Hepatitis C virus was the least prevalent among blood donors over all regions, which reflected the prevalence HCV in the general population. Namibia has 13 regions however only 9 nine regions were used for this study. This is because the NAMBTS only collects blood from these nine regions, excluding the other four which are, Okavango, Caprivi, Omusati and Ohangwena region. The NAMBTS can use the findings of this study to evaluate the health and safety of the blood donors in order to institute improvements that ensure blood safety. It is important for NAMBTS to consider including blood donors from other regions especially the rural population as this might increase the blood donor population, thus increasing blood for the organization.

It also showed that new donors are often the ones that contribute to the bulk of the donations that are positive for TTIs. Regular donors remain an important source of safe blood for Namibia.

### Limitations

The study mainly focused on the donations for only one year. A more comprehensive analysis of the donor characteristics would have been obtained if the data was analyzed for several years. The presence of elite controllers within a donor population is often possible if data is from a much larger population and is from a longer period. It is often preferred to analyze data comprising only one donation per person per year for easy of comparison with other studies. However categorization of data based on type of donor in this study gave some indication of the source of most TTIs.

## Conclusion

The findings of this study show a significantly low prevalence of the four TTIs in this population. This is an indication that blood donors in Namibia are relatively healthy especially the regular donors. The selection criteria for donors and use of a combination of screening methods improve selection of donors who provide safe blood. The findings of this study may not reflect to true prevalence of these infections in the general population as those who are aware that they are HIV positive do not come forward to donate. Even though the prevalence of these TTIs are very low among the blood donor population, it is still very necessary for each unit of blood collected to go through a strict screening process because the infections are still present albeit in very small numbers.

## Competing interests

The authors declare that they have no competing interests.

## Authors’ contributions

RTM contributed to supervision, data analysis and write-up. IC contributed to the laboratory testing, retrieval of data and write-up. ES contributed to the study design, analysis and write-up. MM contributed to the supervision of the work and write-up. All authors participated, read and approved the final manuscript.

## Pre-publication history

The pre-publication history for this paper can be accessed here:

http://www.biomedcentral.com/1471-2458/14/424/prepub
